# Changes of adolescent sleep patterns during the COVID-19 pandemic

**DOI:** 10.11606/s1518-8787.2024058005866

**Published:** 2024-06-17

**Authors:** Mariana Otero Xavier, Jessica Mayumi Maruyama, Iná S. Santos, Luciana Tovo-Rodrigues, Aluísio J. D. Barros, Alicia Matijasevich

**Affiliations:** I Universidade de São Paulo Faculdade de Medicina Departamento de Medicina Preventiva São Paulo SP Brasil Universidade de São Paulo. Faculdade de Medicina. Departamento de Medicina Preventiva. São Paulo,SP, Brasil; II Universidade Presbiteriana Mackenzie Programa de Pós-Graduação em Ciências do Desenvolvimento Humano São Paulo SP Brasil Universidade Presbiteriana Mackenzie. Programa de Pós-Graduação em Ciências do Desenvolvimento Humano. São Paulo, SP, Brasil; III Universidade Federal de Pelotas Faculdade de Medicina Programa de Pós-Graduação em Epidemiologia Pelotas RS Brasil Universidade Federal de Pelotas. Faculdade de Medicina. Programa de Pós-Graduação em Epidemiologia. Pelotas, RS, Brasil

**Keywords:** COVID-19, Adolescent, Sleep, Longitudinal Studies

## Abstract

**OBJECTIVE:**

The COVID-19 pandemic has raised numerous concerns regarding its effects on individuals’ health and lifestyle. We aim to analyze potential changes in adolescent sleep patterns from before and during the pandemic and identify specific predictors of changes.

**METHODS:**

A subgroup of adolescents from a population-based birth cohort from Pelotas, Brazil, was assessed pre-pandemic (T_1_, November-2019 to March-2020) and peri-pandemic (T_2_, August-2021 to December-2021) in in-person interviews (n = 1,949). Sleep parameters, including sleep duration and latency time on workdays and free days, as well as social jetlag (SJL), were assessed using the Munich ChronoType Questionnaire (MCTQ). Socio-demographic, pre-pandemic, and pandemic-related predictors were analyzed. Changes in sleep parameters from T1 to T2 were estimated by multivariate latent change score modeling.

**RESULTS:**

The latent change factor shows a significant mean increase in workday sleep duration (M = 0.334, p < 0.001), workday sleep latency (M = 0.029, p = 0.002), and free day sleep latency (M = 0.021, p = 0.034), and a decreased in SJL (M = −0.758, p < 0.001) during the pandemic. Female adolescents presented higher increases in workday sleep duration. Adolescents who adopted a stricter social distancing level during the pandemic presented greater increases in workday sleep duration and smaller reductions in SJL. Self-evaluated insomnia during the pandemic predicted lower increases in workday and free day sleep duration and higher increases in workday and free day sleep latency.

**CONCLUSION:**

The COVID-19 outbreak brought certain advantages regarding increased sleep duration and reduced SJL. However, the observed increase in sleep latency and the influence of self-reported insomnia could be related to psychological distress inherent to the pandemic.

## INTRODUCTION

Sleep is a physiological state characterized by body rest and reduced consciousness, playing a critical role in several functions, including the effective functioning of the immune system, consolidation of memory, and proper growth and development^1^. Sleep supports learning, attention, cognition, and mental health during adolescence^2^. Due to biological and environmental factors, adolescence is a particularly vulnerable period concerning sleeping habits since it is related to decreased sleep duration, late bedtimes, and increased daytime sleepiness^2^. These sleep patterns contribute to higher levels of social jetlag (SJL) in adolescents, which can be understood as a chronic pattern of desynchronization between biological and social clocks due to a misalignment of weekday obligations (e.g., school starting time)^3^.

In December 2019, the first case of the new coronavirus disease (COVID-19) was registered. Governments addressed measures to contain the spread of contagion, causing sudden changes in lifestyle and work habits. Some studies showed that health-related behaviors of children and adolescents, including physical activity, screen time, and eating behaviors, were negatively affected^4,5^.

The impact on sleep patterns yielded various conclusions^5^. Research conducted during the COVID-19 pandemic revealed extended sleep duration compared to pre-pandemic times^8^. The authors suggested that the absence of commuting to school and the ability to wake up later for online classes during the pandemic allowed adolescents to align their sleep patterns with their natural biological rhythms, as opposed to societal routines. However, despite studies indicating longer sleep durations among children and adolescents, they also noted later bedtimes and poorer sleep quality during the pandemic^5^.

The literature regarding adolescents’ sleep during the COVID-19 pandemic offers important contributions. However, in some studies, comparisons were not with the same group of individuals^8,12^, or there was no comparison group (e.g., before and during the pandemic)^13^. Most studies were cross-sectional and/or used small or non-representative samples^10,11,15^. Several studies were predominantly from online research and retrospectively asked about sleep parameters^14,16^, suggesting a questionable quality of the inferences and subjected participants to recall bias.

Considering the impacts of the COVID-19 pandemic and differences in prevention measures according to country, government responses, and sociodemographic characteristics of the population, it is crucial to conduct studies in different sociocultural contexts for developing potential intervention strategies for similar situations.

Using data from a Brazilian population-based cohort, we aim to examine potential changes in sleep patterns, including sleep duration, sleep latency during workdays and free days, and SJL, among adolescents from the pre-pandemic and the peri-pandemic assessments. We hypothesized that sleep duration would increase during the pandemic, whereas SJL would decrease. Given that the examination of predictors of changes was limited in previous research, we tested whether the changes vary according to sociodemographic, pre-pandemic, and pandemic-related predictors. Regarding our second objective, our hypothesis was that pandemic predictors, such as adopting higher levels of social distancing and having worst perception towards the pandemic, would be the most strongly associated predictors of changes.

## METHODS

### Participants and Procedure

In 2004, all women whose children were born in Pelotas were invited to participate in a longitudinal study. The original cohort consisted of 4,231 newborns from all hospitals in the city, corresponding to 99.2% of the births that year. The mothers were interviewed regarding their socioeconomic, demographic, and reproductive characteristics, and their children were examined after delivery (perinatal study). Mothers and children were also interviewed at their homes at (SD) 3.0 (0.1), 11.9 (0.2), 23.9 (0.4), and 49.5 (1.7) months post-delivery, and at a research clinic at 6.8 (0.3), 11.0 (0.3), and 15.7 (0.2) years post-delivery. Further methodological study details can be found elsewhere^20^.

In this study, data were obtained at two timepoints. The first timepoint (T_1_), dated to November 2019, was the pre-pandemic follow-up, corresponding to the 15-year follow-up (mean age of 15.7 years, SD = 0.2). A total of 1,949 adolescents and their mothers (48.5% of the original cohort) were included. Data collection at the research clinic was interrupted due to social distancing measures imposed by the COVID-19 pandemic (March 2020). The second timepoint (T_2_) was the peri-pandemic follow-up aiming to reassess the same subsample of 1,949 participants, in which pandemic effects were examined by comparing outcomes from immediately before and during the pandemic. The peri-pandemic assessment occurred in the participants’ households from August to December 2021, and accounted for 1,826 adolescents (mean age of 17.4 years, SD = 0.2) and their mothers, corresponding to 89.3% of the target population. Considering that three deaths were identified between T_1_ and T_2_, the 1,826 adolescents evaluated in 2021 represented a retention rate of 93.8%.

### Sleep Parameters

The study outcomes were collected at T_1_ and T_2_, including sleep duration, sleep latency, and absolute SJL. All parameters were assessed using the Munich ChronoType Questionnaire (MCTQ)^21^, referring to the 30 days before the interview. Adolescents were asked about their sleep times separately for days of activities (workdays - including school/academic or work activities) and free days. Sleep duration was defined as the difference between sleep end and sleep onset (when the adolescent was ready to fall asleep + sleep latency). Sleep duration of fewer than 3h or greater than 13h on workdays, and sleep duration of fewer than 3h on free days were considered implausible and excluded from the analysis.

Sleep latency was defined as the amount of time taken to fall asleep. The following question was asked: “*How many minutes do you take to fall asleep?*”. The absolute SJL was defined as the difference between the midsleep point  [sleep onset + (sleep duration/2)]  on free days and the midsleep point on workdays^3^, as follows: 


 SJL = (Midsleep point on free days) − (Midsleep point on workdays )

### Sociodemographic and Pre-pandemic (T1) Predictors of Change

The sociodemographic predictors were assessed at birth and included family income in the month prior to delivery (quintiles), sex at birth (male/female), maternal schooling (completed years of formal education), maternal self-reported skin-color (White or Black/Mixed-race), marital status (mothers living with a partner; yes/no), and maternal age at childbirth. Pre-pandemic predictors were assessed in the 15-year follow-up and included maternal working status (mothers were asked if they were currently working), and maternal depressive symptoms were assessed using the Edinburgh Postnatal Depression Scale (EPDS)^22^.

Caffeine consumption and screen time were assessed during the pre-pandemic period. A series of questions about the consumption of coffee and yerba mate (a typical hot beverage consumed in southern Brazil) was used to evaluate caffeine intake. The daily frequency of consumption was obtained for each source of caffeine. Data on type of coffee (filtered or instant), preparation, concentration (strong, medium, or weak), and quantity consumed per day were also collected, considering recipient size used for drinking coffee (180mL cup; 50mL small cup; 150mL glass; 200mL glass; and 190ml mug). The estimated caffeine content from coffee and yerba mate was obtained following the methods described in a previous study^23^. Daily caffeine consumption from both sources was added and divided by seven to obtain the daily average consumption in a week.

Regarding screen time, adolescents were asked about the time spent in watching television, using the smartphone/tablet, playing video games, and using the computer during leisure time on Sundays (as a proxy of weekends) and on a regular weekday. Time spent on each of gadget was multiplied by two when asked about Sundays and by five when asked about regular weekdays. The sum of which was divided by seven to obtain the average daily number of hours per week in front of an electronic screen media. These values were added to obtain the total screen time. Daily average screen times of more than 15 hours were considered implausible and excluded from the analysis.

### Pandemic-related Predictors of Change

At T_2_, pandemic-related variables that might be associated with the changes in sleep parameters were assessed. The questions included: level of adhesion to social distancing (classified as “strict social distancing,” “moderate social distancing,” and “low/no social distancing”); perceived effect of the pandemic (“How much have you been affected by the pandemic and/or social distancing measures?”, categorized as “not affected at all,” “affected a little,” “moderately affected,” and “affected a lot”); subjective evaluation of screen time during the pandemic (“I spent too much time using my smartphone/computer/other devices and this was not good for me,” yes/no); self-evaluated insomnia/hypersomnia during the pandemic (“I experienced insomnia during the pandemic” and “I felt more sleepy during the pandemic,” yes/no). Screen time was collected and analyzed the same way as in T_1_.

### Data Analysis

**Figure 1:**
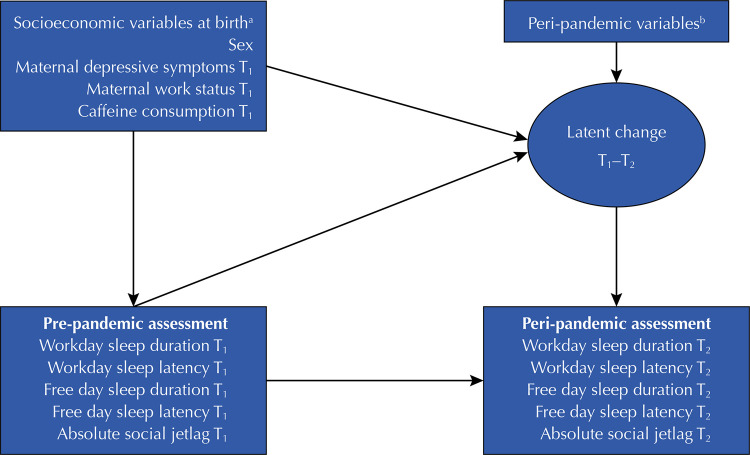
Latent change score models for workday sleep duration, workday sleep latency, free day sleep duration, free day sleep latency, and absolute social jetlag from the pre-pandemic (T1) to the peri-pandemic assessments (T2).

^a^ Socioeconomic variable measured at birth were maternal schooling, family income, maternal skin-color, maternal age, and marital status; ^b^ Peri-pandemic variables were screen time during the pandemic, adopted social distancing levels, perceived impact of the pandemic, subjective evaluation of screen time use, self-evaluation of insomnia during the pandemic, and self-evaluation of hypersomnia during the pandemic. Adolescent age was also included in all models.

For a proper estimate of the changes in adolescent sleep parameters at T_2_ compared to T_1_, multivariate latent change score (LCS) modeling approach was used, which provides robust estimates of changes over time^24^. As shown in Figure 1, changes in each sleep measure were modelled as five parallel processes, including workday sleep duration, workday sleep latency, free day sleep duration, free day sleep latency, and absolute SJL.

Autoregressive paths from T_1_ measures to the latent change score factors were included to examine the proportional changes (i.e., the extent to which the latent change score factors depend on the initial sleep levels). A multivariate latent change score model was constructed to investigate the predictors of sleep measures at T_1_ and the latent change scores. Adolescents’ age was also included in all models to account for its possible effect on the initial levels and latent change scores. Latent change score factor of all sleep measures was correlated with each other to identify whether change processes were interrelated. The analyses were conducted in Mplus 8.4 software program, adopting maximum likelihood estimation with robust standard errors (MLR)^25^. Missingness is accounted for with full information maximum likelihood estimation (FIML), assuming responses are missing at random (MAR) including a final sample of 1,949 adolescents. The model fit (good model fit cut-off in parenthesis) in multivariate latent change analysis was evaluated by using the Comparative Fit Index (CFI ≥ 0.90), the Tucker-Lewis Index (TLI ≥ 0.90), the Root Mean Square Error of Approximation (RMSEA < 0.08), and the Standardized Root Mean Square Residual (SRMR < 0.08)^26^.

### Ethics

The 2004 Pelotas (Brazil) Birth Cohort Study was approved by the Research Ethics Committee of the Medical School of the Federal University of Pelotas. All main caregivers and adolescents signed informed consent forms before data collection. The study was also approved by the Ethics Committee for Analysis of Research Projects (CAPPesq) of the Hospital das Clínicas of the School of Medicine of the Universidade de São Paulo (USP) (Research protocol Nº 4.951.457).

## RESULTS

### Descriptive statistics

Table 1 presents sociodemographic characteristics of the study sample. The participants have mothers with more years of education, not single at birth, and higher family income when compared to those not interviewed. No statistical differences were found between the T_1_ and T_2_ samples regarding socioeconomic factors (Table 1).

**Table 1:** Distribution of the adolescents interviewed in the pre-pandemic (T1) and peri-pandemic assessments (T2) from the 2004 Pelotas (Brazil) Birth Cohort Study.

Characteristic	Not interviewed n = 2,372	Pre-pandemic assessment (T_1_) n = 1,949	Peri-pandemic assessment (T_2_) n = 1,805
		
	n (%)	n (%)	n (%)
Maternal schooling at birth (years)			
Mean (SD)	7.90 (3.51)^a^	8.30 (3.41)	8.31 (3.41)
Range	0–18	0–17	0–17
Family income at birth (quintiles)			
1st quintile (poorest)	497 (22.51)^a^	374 (18.51)	327 (18.12)
2nd to 5th quintile	1,713 (77.49)	1647 (81.49)	1,478 (81.88)
Living with a partner at childbirth			
Yes	1,818 (82.34)^a^	1,718 (85.01)	1,533 (84.93)
No	390 (17.66)	303 (14.99)	272 (15.07)
Maternal skin-color			
White	1,599 (72.42)	1,489 (73.68)	1,325 (73.41)
Black or Mixed race	609 (27.58)	532 (26.32)	480 (26.59)
Maternal age at birth			
Mean (SD)	25.66 (6.68)	26.52 (6.92)	26.71 (6.99)
Range	13–46	13–45	13–46
Adolescent’s sex			
Male	1,161 (52.53)	1,034 (51.16)	917 (50.80)
Female	1,049 (47.47)	987 (48.12)	888 (49.20)
Adolescents’ age			
Mean (SD)	-	15.69 (0.19)	17.41 (0.26)
Range		15.01–16.15	16.7–17.9

^a^ p < 0.05 for the difference between the pre-pandemic sample (T_1_; n = 1,949) and the sample not included in the current study (n = 2,372). There were no statistically differences in socioeconomic variables between the pre-pandemic (T_1_) and peri-pandemic samples (T_2_).

### Latent change score modelling

The multivariate latent change model yielded a good model fit: χ2 = 213.991, *df* = 62, CFI/TLI = 0.948/0.813, RMSEA (90%CI) = 0.036 (0.031, 0.042), SRMR = 0.020.

The mean of the latent change factor was positive and significant for workday sleep duration (*M* = 0.334, SE = 0.057, p < 0.001), workday sleep latency (*M* = 0.029, SE = 0.009, p = 0.002), and free day sleep latency (*M* = 0.021, SE = 0.010, p = 0.034) (Table 2). The mean of the latent change factor was negative and significant for SJL (*M* = −0.758, SE = 0.045, p < 0.001). The mean of the latent change factor for free day sleep duration was not significant (*M* = −0.039, SE = 0.048, p = 0.415), indicating that this measure did not change from T_1_ to T_2_. The variances for all latent change scores were significant, suggesting interindividual differences in the sleep measures by different adolescents in the study sample (Table 2). All coefficients of the proportional change were significant and negative, showing that higher sleep measures at T_1_ were associated with lower latent changes during the study period (Table 2).

**Table 2:** Latent change scores, individual variability, and proportional change on workday sleep duration, workday sleep latency, free day sleep duration, free day sleep latency, and absolute social jetlag from pre-pandemic wave (T1) to peri-pandemic wave (T2) among adolescents from the 2004 Pelotas (Brazil) Birth Cohort Study.

	Latent change scores (LCS; T_1_ – T_2_)		Individual variance		Proportional change	
		
	M (SE)	p-value	σ ^2^	p-value	b	p-value
Workdays						
Sleep duration	0.334 (0.057)	<0.001	5.059	<0.001	−2.697	< 0.001
Sleep latency	0.029 (0.009)	0.002	0.138	<0.001	−0.057	< 0.001
Free days						
Sleep duration	−0.039 (0.048)	0.415	3.875	<0.001	−2.159	< 0.001
Sleep latency	0.021 (0.010)	0.034	0.168	<0.001	−0.094	< 0.001
Absolute social jetlag	−0.758 (0.045)	<0.001	2.958	<0.001	−1.569	< 0.001

SE: standard error

Note: Unstandardized coefficients are shown. Maximum likelihood robust estimator was used, with full maximum information likelihood for handling missing data.

Latent change scores show the mean increase or decrease of each sleep measures between pre-pandemic and peri-pandemic waves, modelled as a latent variable; Individual variance (σ^2^) capture the extent to which individuals differ in the change they manifest over time; Proportional change shows the extent to which the LCS are related to pre-pandemic scores.

The correlations between the latent change score factors were all significant and varied from very weak to strong values (−0.013 to 0.612). Changes in workday sleep latency were strongly correlated to changes in free day sleep latency (r = 0.612, p < 0.001), while changes in workday sleep duration were moderately correlated with changes in free day sleep duration (r = −0.124, p < 0.001). In addition, changes in workday sleep duration were moderately correlated with changes in SJL (r = −0.375, p < 0.001) (Data not shown).

Table 3 shows the predictors of sleep measures at T_1_. Higher maternal schooling predicted reduced workday sleep duration and a higher SJL. A higher family income also predicted a higher SJL. Female adolescents presented a higher free day sleep duration and latency and a lower SJL. A higher caffeine consumption at T_1_ was associated with shorter sleep duration only during the free days. A greater screen time predicted shorter sleep duration, higher sleep latency in the workday, and higher SJL (Table 3).

**Table 3:** Predictors of workday sleep duration, workday sleep latency, free day sleep duration, free day sleep latency, and absolute social jetlag in the pre-pandemic wave among adolescents from the 2004 Pelotas (Brazil) Birth Cohort.

Sleep measures at T_1_					

	Workdays		Free days		Absolute social jetlag
	
	Sleep duration	Sleep latency	Sleep duration	Sleep latency	
				
	*β* (SE)	*β* (SE)	*β* (SE)	*β* (SE)	*β* (SE)
Maternal schooling (years)	−0.090 (0.026)^c^	−0.018 (0.025)	−0.027 (0.028)	−0.033 (0.037)	0.071 (0.027)^b^
Family income (quintiles)	−0.038 (0.027)	0.015 (0.029)	0.008 (0.028)	0.014 (0.026)	0.083 (0.028)^b^
Single mother	0.012 (0.025)	0.027 (0.023)	0.011 (0.026)	0.035 (0.025)	−0.050 (0.027)^a^
Black/Mixed-race maternal skin color	0.001 (0.026)	−0.032 (0.023)	−0.047 (0.025)^a^	−0.003 (0.026)	0.020 (0.024)
Maternal age	−0.003 (0.024)	0.014 (0.024)	−0.006 (0.024)	0.031 (0.026)	−0.044 (0.024)^a^
Female adolescents	0.011 (0.024)	0.029 (0.025)	0.086 (0.024)^c^	0.074 (0.023)^c^	−0.081 (0.024)^c^
Adolescent age (T_1_)	0.022 (0.022)	0.017 (0.025)	0.001 (0.023)	0.034 (0.021)^a^	−0.045 (0.023)^b^
Maternal work status (T_1_)	−0.030 (0.027)	0.001 (0.026)	0.016 (0.027)	0.021 (0.025)	0.049 (0.027)^a^
Maternal depressive symptoms (T_1_)	0.028 (0.028)	0.007 (0.025)	0.047 (0.027)^a^	0.015 (0.025)	0.001 (0.026)
Caffeine consumption (T_1_)	0.027 (0.024)	−0.044 (0.020)^b^	−0.091 (0.028)^b^	−0.028 (0.028)	−0.027 (0.025)
Screen time (T_1_)	−0.064 (0.020)^b^	0.040 (0.021)^b^	−0.035 (0.020)^a^	0.033 (0.019)^a^	0.041 (0.021)^b^

SE: standard error

Note: Standardized coefficients are shown. Maximum likelihood robust estimator was used, with full maximum information likelihood for handling missing data. All models included adolescent age at the assessment.

^a^ *p* < 0.100; ^b^ *p* < 0.05; ^c^ *p* < 0.001

Table 4 shows the predictors for the latent change scores from T_1_ to T_2_. A negative coefficient means that higher levels of the predictor are associated with smaller changes in sleep measures. Female adolescents presented a greater increase in workday sleep duration. Adolescents from mothers working before the pandemic had smaller increase in workday sleep latency. Adopting a stricter social distancing level during the pandemic was a positive predictor of change in workday sleep duration and a negative predictor of change for SJL. The self-evaluated insomnia during the pandemic was the most important predictor of change for all sleep measures except for SJL: it was a negative predictor for changes in workday and free day sleep duration and a positive predictor of change for workday and free day sleep latency (Table 4).

**Table 4:** Predictors of workday sleep duration, workday sleep latency, free day sleep duration, free day sleep latency, and absolute social jetlag in the peri-pandemic wave among adolescents from the 2004 Pelotas (Brazil) Birth Cohort.

Latent change score models for sleep measures between T_1_ and T_2_					

	Workdays		Free days		Absolute social jetlag
	
	Sleep duration	Sleep latency	Sleep duration	Sleep latency	
				
	*β* (SE)	*β* (SE)	*β* (SE)	*β* (SE)	*β* (SE)
Socioeconomic and T_1_ predictors					
Maternal schooling	0.028 (0.028)	0.011 (0.029)	0.051 (0.029)^a^	−0.018 (0.032)	0.030 (0.029)
Family income	0.011 (0.030)	−0.013 (0.028)	−0.043 (0.028)	−0.016 (0.027)	−0.043 (0.030)
Single mother	−0.024 (0.028)	−0.007 (0.022)	0.001 (0.026)	−0.003 (0.019)	0.025 (0.030)
Black/Mixed-race maternal skin color	0.033 (0.028)	0.003 (0.024)	0.036 (0.026)	−0.026 (0.025)	0.014 (0.026)
Maternal age	−0.010 (0.026)	0.022 (0.026)	0.019 (0.024)	−0.006 (0.028)	0.023 (0.025)
Female adolescents	0.087 (0.027)^c^	−0.018 (0.026)	0.020 (0.025)	−0.027 (0.025)	−0.037 (0.026)
Maternal work status (T_1_)	−0.012 (0.028)	−0.047 (0.027)^b^	0.011 (0.027)	−0.035 (0.025)	0.001 (0.028)
Maternal depressive symptoms (T_1_)	−0.021 (0.029)	−0.006 (0.026)	−0.017 (0.027)	0.001 (0.026)	0.009 (0.027)
Caffeine consumption (T_1_)	−0.013 (0.029)	0.044 (0.028)	0.032 (0.030)	0.007 (0.027)	0.047 (0.029)^a^
Peri-pandemic predictors					
Adolescent age (T_2_)	−0.039 (0.022)^a^	−0.047 (0.025)^a^	−0.014 (0.023)	−0.052 (0.022^)b^	0.041 (0.024)^a^
Social distancing level	0.050 (0.020)^b^	0.002 (0.021)	0.008 (0.019)	0.023 (0.019)	−0.107 (0.022)^c^
Perceived impacts of the pandemic	−0.026 (0.020)	0.001 (0.024)	−0.018 (0.021)	−0.003 (0.020)	−0.003 (0.022)
Screen time (T_2_)	−0.036 (0.022)^a^	0.014 (0.027)	−0.040 (0.020)^a^	0.022 (0.028)	0.007 (0.023)
Subjective evaluation of screen time	−0.010 (0.021)	0.007 (0.020)	−0.017 (0.021)	0.006 (0.019)	−0.020 (0.022)
Self-evaluated insomnia during the pandemic	−0.069 (0.021)^c^	0.177 (0.021)^c^	−0.048 (0.021)^b^	0.148 (0.019)^c^	0.012 (0.023)
Self-evaluated hypersomnia during the pandemic	0.022 (0.021)	0.016 (0.021)	0.009 (0.020)	0.031 (0.018)^a^	0.024 (0.022)

SE: standard error

Note: Standardized coefficients are shown. Maximum likelihood robust estimator was used, with full maximum information likelihood for handling missing data. All models included adolescent age at the assessment.

^a^ *p* < 0.100; ^b^ *p* < 0.05; ^c^ *p* < 0.001

## DISCUSSION

We found a slight increase in the mean workday sleep duration and sleep latency when comparing T_2_ to T_1_, while SJL decreased during the pandemic. These findings were consistent with the literature, as previous studies found similar results^5^. Adolescents tend to start sleeping later at night and prefer to sleep longer in the morning^2^, which was possible during online classes since students did not need to commute to school. We highlight that adolescents’ biological changes, not related to the pandemic, offer another possibility of explanation, given the long period that elapsed between the two assessments and the natural tendency to modify the circadian rhythm throughout adolescence^2^.

Other studies identified that the increase in sleep duration occurred mostly during weekdays (workdays) compared to weekends (free days)^9,18^. Most schools usually start very early in the morning for this age group, and it is common for many teenagers to suffer from SJL. This circadian misalignment has been associated with increased health risks and health-impairing behavior.^4^ Most activities during the pandemic (e.g., school and extracurricular activities) were conducted on electronic-based platforms, offering adolescents more flexibility regarding sleep schedules. On weekends, most adolescents were free to follow their sleep schedules, so it is plausible that there were no changes. These findings point to the importance of adolescent’s adherence to their internal biological clock and allows policymakers to help reduce adolescent’s SJL outside of pandemic circumstances.

We found that longer sleep durations and longer sleep latencies in T_1_ presented fewer changes in T_2_. Even with an increase in sleep duration, adolescents reported an average sleep duration on workdays lower than the recommended amount of sleep (8 to 10 hours) for this age group. Short sleep duration is associated with poor academic performance and poor health behaviors, while sufficient sleep is crucial to adolescent daytime functioning, health, and well-being^27^.

The increased sleep latency during the pandemic could be related to at least three reasons. First, the difficulty in falling asleep may be caused by psychological distress due to the pandemic^7^. Despite COVID-19 offering more health risks for middle-aged and older adults, the emotional well-being of younger people was also affected. Fear of infection, social distancing, changes in family financial situation, and uncertainty about the future significantly affected Brazilian adolescents^28^. Second, the considerable increase in the use of screen time during the pandemic related to home-schooling and other online activities could impair sleep, as it may increase blue light exposure, which can suppress natural melatonin production and disrupt the circadian rhythm, leading to delayed sleep phase and difficulties to relax^29^. Third, sleep latency is influenced by activities conducted during the day, such as physical activity practice. Adolescents reduced physical activity practice during the pandemic and replaced it with more time spent in sedentary behavior^4,5^. Additionally, remote activities reduced adolescents’ sunlight exposure, also reducing the presence of *zeitgebers* (elements capable of regulating the biological clock), causing a mismatch in body-clocks^7^. In Brazil, an online survey with 9,470 adolescents from public and private schools showed that adolescents were negatively emotionally affected by the uncertainty of the COVID-19 pandemic’s progression parallel to unhealthy behaviors and isolation from social circles^30^.

Analysis of specific predictors of changes in adolescent’s sleep parameters revealed a more pronounced increase in sleep duration among female adolescents than their male counterparts. Other studies found distinct results for boys and girls^13,19^, including a study about sleep quality during the COVID-19 pandemic with 153 Brazilian adolescents, which found that the pandemic affected more females than males (girls presented worse sleep quality and more sleep disorders, as well as anxiety and sadness)^13^. A possible explanation could be associated with the fact that, historically, females are frequently more connected and more perceptive about their health issues.

In our study, we verified that adolescents whose mothers were working before the pandemic presented smaller increases in workday sleep latency. Sleep quality, such as lower latency time, few night awakenings, and high sleep efficiency (referring to the time in bed sleeping), are related to psychosocial factors^31^. In adolescence, family environment, including economic stability and a sense of security, are important positive factors for healthful sleep^31,32^. For adolescents whose mothers were out of work, the situation may have become even more difficult during the pandemic, causing concerns and insecurity in their sleep. However, most mothers who already had a job managed to keep it, which may have given their children more emotional stability, presenting smaller increases in latency time. In this context, it is important to point out that self-evaluated insomnia during the pandemic was the most important predictor of change for all sleep measures except for SJL. Adolescents who reported insomnia during the pandemic presented a smaller increase in sleep duration and higher increases in sleep latency.

### Strengths and Limitations

Strengths of this study include the longitudinal design with the opportunity to compare data obtained shortly before and during COVID-19 in the same group of individuals. To our knowledge, this was the first longitudinal study using a population-based sample with high social diversity, which investigated pre-pandemic and pandemic-related predictors associated with changes in sleep parameters. The use of latent change score modelling as a method of analysis contributed to the robustness of the study, specifically tailored to overcome various weaknesses of more traditional approaches^24^. With this methodology, we adjusted for proportional changes (how much the degree of change from T_1_ to T_2_ is influenced by the T_1_ level presented by the individual).

Regarding limitations, sleep measures were self-reported, and we suggest further studies to incorporate more objective methods such as actigraphy. Despite the longitudinal data collection, it is impossible to attribute the detected changes only to the effects of the COVID-19 pandemic. The time between the two assessments of more than one year is a relatively long period, and many changes in interindividual factors, including lifestyle habits and family routines, may have occurred during such time regardless of the pandemic. Lastly, the 15-year follow-up of the cohort had to be interrupted due to the COVID-19 pandemic, resulting in a loss to follow-up of nearly 50% from the original cohort. Although there were few differences between the original cohort and the study sample, selection bias cannot be excluded.

In our study, the COVID-19 pandemic was associated with advantageous changes in some sleep parameters, such as increased sleep duration during workdays and reduced SJL, emphasizing probable advantages of longer sleep times due to flexibility in schedules related to social retainment. However, sleep latency slightly increased, which can be considered a poor sleep quality parameter associated with sleep onset difficulties. These difficulties were probably related to pandemic-induced psychological distress, confirmed by the observed influence of self-perceived insomnia.

Accumulating knowledge about the effects of the pandemic on populations, especially young individuals, is extremely important to detect possible longer-term repercussions, determine specific protective and risk factors, develop timely preventive and interventional strategies, and prepare society for future similar events. Finally, we encourage parents and adolescents to learn and adopt sleep hygiene practices to promote and maintain optimal sleep quality during this critical developmental time frame.
